# Indications and efficacy of canakinumab in Familial Mediterranean Fever: report of three cases

**DOI:** 10.1186/1546-0096-12-S1-P253

**Published:** 2014-09-17

**Authors:** Maria Tsinti, Elena Tsitsami

**Affiliations:** 1 Children's Hospital "Aghia Sofia", Athens, Greece; 2 1st Department of Pediatrics, University of Athens, Athens, Greece

## Introduction

Evidence from case reports/series, one controlled study, and one phase II, open-label, single-arm study supports IL-1β blockage as a potential treatment for colchicine resistant Familial Mediterranean Fever (FMF).

## Objectives

We report three cases of FMF successfully treated with canakinumab administered due to unresponsiveness or intolerance to colchicine.

## Methods

Case series.

## Results

The first case is son of a mother with FMF. He was diagnosed for FMF at the age of 3.5 years and since then he was successfully treated with colchicine. He was heterozygous for the V726A mutation and carrier of polymorphisms in exon 2 of the *MEFV* gene. The patient referred to our Unit at the age of 11 years because, during the last 5 years, he suffered recurrent respiratory infections (>10/year) including four pneumonias. Laboratory investigations revealed lymphopenia (lymphocytes 800/μL), low serum IgG, IgA and IgM levels but with normal specific antibody responses, and normal CRP and SAA levels. We attributed lymphopenia and low immunoglobulin levels to colchicine. Decreasing of colchicine dosage resulted in the recurrence of FMF symptoms with elevation of CRP and SAA levels. However, lymphopenia and hypogammaglobulinemia persisted even after the discontinuation of colchicine for 2 months. After that, the administration of canakinumab (4 mg/kg/6 weeks) was attempted. FMF symptoms waned immediately, while lymphocyte count and immunoglobulin levels were normalized after the forth dose. Only one infectious episode was appeared during the 6-month period of canakinumab administration.

The second patient presented at the age of 13 years with severe fatigue and mild but constant elevation of CRP, ESR and SAA since birth. Detailed examination revealed periodic arthralgias in the ankles and sensorineural deafness, beginning six months ago. Genetic analysis revealed heterozygosity for the V726A mutation in the *MEFV* gene but no mutations in exon 3 of the *NLPR3*. Colchicine (1 mg daily) was initiated, with improvement of symptoms but without change of inflammatory markers after 6 months of administration. Administration of canakinumab (150 mg/4 weeks) led to normalization of inflammatory markers for the first time since his birth. The patient remains asymptomatic after 4 doses of canakinumab.

The third case is a teenager who had been diagnosed with FMF at the age of 4 due to typical symptoms and homozygosity for the M694V mutation. The disease had been kept into remission with colchicine until the age of 14 when he presented every 3 weeks severe flares (mild fever but severe chest pain, arthralgias and fatigue followed by loss of weight), unresponsive to maximum doses colchicine. Canakinumab (150 mg/6 weeks) was initiated and led into remission. In a period of 2 years the patient presented 3 mild episodes.

No adverse effects were observed in anyone of the patients during the whole periods of canakinumab administration.

## Conclusion

The reported cases indicate that canakinumab is effective and safe for the treatment of FMF patients resistant to colchicine as well as of those presenting adverse effects of the drug.

## Disclosure of interest

None declared.

